# Adverse Outcomes after Major Surgery in Patients with Pressure Ulcer: A Nationwide Population-Based Retrospective Cohort Study

**DOI:** 10.1371/journal.pone.0127731

**Published:** 2015-05-22

**Authors:** Chia-Lun Chou, Woan-Ruoh Lee, Chun-Chieh Yeh, Chun-Chuan Shih, Ta-Liang Chen, Chien-Chang Liao

**Affiliations:** 1 Department of Dermatology, School of Medicine, College of Medicine, Taipei Medical University, Taipei, Taiwan; 2 Department of Dermatology, Shuang Ho Hospital, Taipei Medical University, New Taipei City, Taiwan; 3 Graduate Institute of Medical Sciences, Taipei Medical University, Taipei, Taiwan; 4 Department of Surgery, China Medical University Hospital, Taichung, Taiwan; 5 Department of Surgery, University of Illinois, Chicago, United States of America; 6 School of Chinese Medicine for Post-Baccalaureate, I-Shou University, Kaohsiung, Taiwan; 7 Department of Anesthesiology, Taipei Medical University Hospital, Taipei, Taiwan; 8 Health Policy Research Center, Taipei Medical University Hospital, Taipei, Taiwan; 9 Department of Anesthesiology, School of Medicine, College of Medicine, Taipei Medical University, Taipei, Taiwan; 10 School of Chinese Medicine, China Medical University, Taichung, Taiwan

## Abstract

**Background:**

Postoperative adverse outcomes in patients with pressure ulcer are not completely understood. This study evaluated the association between preoperative pressure ulcer and adverse events after major surgeries.

**Methods:**

Using reimbursement claims from Taiwan’s National Health Insurance Research Database, we conducted a nationwide retrospective cohort study of 17391 patients with preoperative pressure ulcer receiving major surgery in 2008-2010. With a propensity score matching procedure, 17391 surgical patients without pressure ulcer were selected for comparison. Eight major surgical postoperative complications and 30-day postoperative mortality were evaluated among patients with pressure ulcer of varying severity.

**Results:**

Patients with preoperative pressure ulcer had significantly higher risk than controls for postoperative adverse outcomes, including septicemia, pneumonia, stroke, urinary tract infection, and acute renal failure. Surgical patients with pressure ulcer had approximately 1.83-fold risk (95% confidence interval 1.54-2.18) of 30-day postoperative mortality compared with control group. The most significant postoperative mortality was found in those with serious pressure ulcer, such as pressure ulcer with local infection, cellulitis, wound or treatment by change dressing, hospitalized care, debridement or antibiotics. Prolonged hospital or intensive care unit stay and increased medical expenditures were also associated with preoperative pressure ulcer.

**Conclusion:**

This nationwide propensity score-matched retrospective cohort study showed increased postoperative complications and mortality in patients with preoperative pressure ulcer. Our findings suggest the urgency of preventing and managing preoperative pressure ulcer by a multidisciplinary medical team for this specific population.

## Introduction

Pressure ulcer remains a significant problem commonly found in disabled patients who need long-term care. It was estimated by researchers in 2003 and 2013 that between 1.3 and 3 million adults had pressure ulcer in the United States [1,2]. Hospital stays increased nearly 80% between 1993 and 2006 among patients with pressure ulcers, resulting in a remarkable increase in the annual financial burden of the disease [2,3].

Limited mobility due to spinal cord injury, cerebrovascular accident or hip fracture was an important contributing factor in pressure ulcer [4,5]. Diabetes, congestive heart failure, renal dysfunction, chronic obstructive pulmonary disease, progressive neurologic disorder, malnutrition and aging skin were also considered as medical comorbidities associated with pressure ulcer [4,5]. The influences of extrinsic pressure, friction, shear stress and excessive moisture contribute to tissue hypoxia and poor wound healing followed by tissue necrosis [5]. Pressure ulcers may lead to patient distress, poor quality of life and recurrent severe infection; these often increase hospital stay length [3,6], mortality rates [7], and need for long-term health care [3].

Appropriate clinical intervention is recommended for patients receiving surgery with high risk of developing pressure ulcer [8–12]. However, whether preoperative pressure ulcer is associated with postoperative adverse outcomes was still unclear.

In this nationwide population-based cohort study, we used Taiwan’s National Health Insurance Research Database to validate postoperative complications and mortality in patients with preoperative pressure ulcers undergoing major surgeries. The impact of various severities of pressure ulcers on surgical adverse events was also analyzed.

## Methods

### Source of data

Research data were obtained from reimbursement claims of the Taiwan National Health Insurance Program, which was implemented in March 1995 and covers more than 99% of the 22.6 million Taiwan residents. The National Health Research Institutes established the National Health Insurance Research Database that records all beneficiaries’ medical services, including inpatient and outpatient demographics, primary and secondary diagnoses, procedures, prescriptions and medical expenditures for public research interest. The validity of this database has been favorably evaluated, and research articles based on it have been accepted in prominent scientific journals worldwide [13–16].

### Ethical approval

Insurance reimbursement claims used in this study were from Taiwan’s National Health Insurance Research Database, which is available for public access. This study was conducted in accordance with the Helsinki Declaration. To protect personal privacy, the electronic database was decoded with patient identifications scrambled for further public access for research. According to National Health Research Institutes regulations, informed consent is not required because of the use of decoded and scrambled patient identifications. However, this study was evaluated and approved by Taiwan’s National Health Research Institutes (NHIRD-103-121). The data are owned by the National Health Insurance Research Database Committee [13–16] and the contact information is available as email: nhird@nhri.org.tw.

### Study design

We examined medical claims and identified 17391 patients aged ≥20 years with preoperative pressure ulcer from 1,499,745 patients who underwent major inpatient surgeries between 2008 and 2010 in Taiwan. These surgeries required general, epidural or spinal anesthesia and hospitalization for more than one day. To identify patients with pressure ulcer, the present study required at least one visit for outpatient or inpatient medical services for a diagnosis of pressure ulcer within the 24-month preoperative period. We matched each surgical patient with pressure ulcer with a randomly selected surgical patient without pressure ulcer by sex, age, type of surgery, type of anesthesia, coexisting medical conditions, operation in teaching hospital or not, and low income or not, and conducted the analysis with a propensity score-matched pair procedure.

### Measures and definition

We identified income status by defining low-income patients as those qualifying for waived medical copayment, because this status is verified by the Bureau of National Health Insurance. Whether the surgery was performed in a teaching hospital and the types of surgery and anesthesia were also recorded. We used the *International Classification of Diseases*, *Ninth Revision*, *Clinical Modification* (ICD-9-CM) to define preoperative medical diseases and postoperative complications. Preoperative pressure ulcer (ICD-9-CM 707.0) was defined as the major exposure. Pre-existing medical conditions that were determined from medical claims for the 24-month preoperative period included hypertension (ICD-9-CM 401–405), mental disorders (ICD-9-CM 290–319), diabetes (ICD-9-CM 250), chronic obstructive pulmonary disease (ICD-9-CM 490–496), stroke (ICD-9-CM 430–438), ischemic heart disease (ICD-9-CM 410–414), congestive heart failure (ICD-9-CM 428), liver cirrhosis (ICD-9-CM 571), hyperlipidemia (ICD-9-CM 272.0, 272.1, and 272.2), spinal cord injury (ICD-9-CM 806), hip fracture (ICD-9-CM 820), and peripheral vascular disease (ICD-9-CM 443). Renal dialysis was defined by administration code (D8, D9). In-hospital 30-day mortality after the index surgery was considered the study’s primary outcome. Eight major surgical postoperative complications were noted, including septicemia (ICD-9-CM 038, 998.5), pneumonia (ICD-9-CM 480–486), stroke (ICD-9-CM 430–438), urinary tract infection (ICD-9-CM 599.0), acute renal failure (ICD-9-CM 584), deep wound infection (ICD-9-CM 958.3), pulmonary embolism (ICD-9-CM 415), postoperative bleeding (ICD-9-CM 998.0, 998.1 and 998.2) and acute myocardial infarction (ICD-9-CM 410) after the index surgery [15,16]. Admission to intensive care unit, prolonged length of stay, and increased medical expenditure after index surgery were analyzed as secondary outcomes. To investigate the severity of pressure ulcer on 30-day postoperative mortality, we identified some characteristics in patients with pressure ulcer: debridement (administration code F48001C, F48002C, F48003C, F48004C, F48006C, F48006C), local infection (ICD-9-CM 686) and cellulitis (ICD-9-CM 681, 682). The use of first-line (amoxicillin, ampicillin, amoxicillin/clavulanic acid, baktar, cefazolin, gentamicin, oxacillin, unasyn) or second-line (clindamycin, linezolid, meropenem, teicoplanin, tigecycline, vancomycin) antibiotics for treating pressure ulcer and related illness within 24 months preoperatively were considered in this study. The cumulative use (i.e. the total quantity) of such antibiotics was categorized into quartiles. The cumulative medical expenditure on antibiotics was also categorized as low (lowest quartile), moderate (second quartile), and high (third quartile and highest quartile).

### Statistical analysis

To reduce confounding errors [16], this study used a propensity score-matched pair procedure to balance the covariates between surgical patients with and without pressure ulcer. We developed a nonparsimonious multivariable logistic regression model to estimate a propensity score for preoperative renal dialysis. Clinical significance guided the initial choice of covariates in this model: sex, age, types of surgery, types of anesthesia, operation in a teaching hospital or not, low-income status, hypertension, mental disorders, diabetes, chronic obstructive pulmonary disease, stroke, ischemic heart disease, congestive heart failure, liver cirrhosis, hyperlipidemia, renal dialysis, and peripheral vascular disease. A structured iterative approach was used to refine this model, with the goal of achieving covariate balance within the matched pairs. We used chi-square tests to measure covariate balance, and p < 0.05 was suggested to represent meaningful covariate imbalance. We matched patients with pressure ulcer to non-pressure ulcer patients using a greedy-matching algorithm with a caliper width of 0.2 SD of the log odds of the estimated propensity score. This method could remove 98% of the bias from measured covariates [16].

Adjusted rate ratios (RRs) with 95% confidence intervals (CIs) for 30-day postoperative complications and mortality between patients with and without pressure ulcer were analyzed with multivariate Poisson regression models by controlling for sex, age, low-income status, operation in a teaching hospital or not, coexisting medical conditions, and types of surgery and anesthesia. We also performed stratification analysis in age, sex and coexisting medical conditions for the association between preoperative pressure ulcer and postoperative mortality. The multivariate Poisson regression analyses were applied to investigate 30-day postoperative mortality associated with severity of pressure ulcer after controlling for sex, age, low-income status, operation in a teaching hospital or not, preoperative coexisting medical conditions, and types of surgery and anesthesia. SAS version 9.1 (SAS Institute Inc., Cary, NC, USA) statistical software was used for data analyses; two-sided p < 0.05 indicated significant differences.

## Results

Table 1 shows demographic characteristics of patients with and without preoperative pressure ulcer who underwent major surgeries. After propensity score matching, there were no significant differences in perioperative characteristics between surgical patients with and without pressure ulcer, including sex, age, types of surgery and anesthesia, operation in a teaching hospital or not, low-income status, hypertension, mental disorders, diabetes, chronic obstructive pulmonary disease, stroke, ischemic heart disease, congestive heart failure, liver cirrhosis, hyperlipidemia, renal dialysis and peripheral vascular disease.

**Table 1:** Characteristics of surgical patients with or without pressure ulcer. *

	No PU (N = 17391)		PU (N = 17391)		
	n	(%)	n	(%)	*P*
Age, years					1.00
20–29	397	(2.3)	397	(2.3)	
30–39	541	(3.1)	541	(3.1)	
40–49	883	(5.1)	883	(5.1)	
50–59	1515	(8.7)	1515	(8.7)	
60–69	2454	(14.1)	2454	(14.1)	
70–79	5845	(33.6)	5845	(33.6)	
80–89	5041	(29.0)	5041	(29.0)	
≥90	715	(4.1)	715	(4.1)	
Sex					1.00
Female	7987	(45.9)	7987	(45.9)	
Male	9404	(54.1)	9404	(54.1)	
Low income	1100	(6.3)	1100	(6.3)	1.00
Urbanization					1.00
Low	5369	(30.9)	5369	(30.9)	
Moderate	4312	(24.8)	4312	(24.8)	
High	4326	(24.9)	4326	(24.9)	
Very high	3384	(19.5)	3384	(19.5)	
Operation in teaching hospital	15780	(90.7)	15780	(90.7)	1.00
Type of surgery					1.00
Skin	1854	(10.7)	1854	(10.7)	
Breast	49	(0.3)	49	(0.3)	
Musculoskeletal	7856	(45.2)	7856	(45.2)	
Respiratory	775	(4.5)	775	(4.5)	
Cardiovascular	927	(5.3)	927	(5.3)	
Digestive	2381	(13.7)	2381	(13.7)	
Kidney, ureter, bladder	1278	(7.4)	1278	(7.4)	
Delivery, CS, abortion	50	(0.3)	50	(0.3)	
Neurosurgery	1679	(9.7)	1679	(9.7)	
Eye	78	(0.5)	78	(0.5)	
Others	464	(2.7)	464	(2.7)	
Type of anesthesia					1.00
General	12085	(69.5)	12085	(69.5)	
Epidural or spinal	5306	(30.5)	5306	(30.5)	
Coexisting medical conditions					
Hypertension	5924	(34.1)	5924	(34.1)	1.00
Mental disorders	5119	(29.4)	5119	(29.4)	1.00
Diabetes	4990	(28.7)	4990	(28.7)	1.00
COPD	3660	(21.1)	3660	(21.1)	1.00
Stroke	2856	(16.4)	2856	(16.4)	1.00
Hip fracture	2104	(12.1)	2104	(12.1)	1.00
Ischemic heart disease	1929	(11.1)	1929	(11.1)	1.00
Congestive heart failure	658	(3.8)	658	(3.8)	1.00
Renal dialysis	526	(3.0)	526	(3.0)	1.00
Spinal cord injury	340	(2.0)	340	(2.0)	1.00
Hyperlipidemia	307	(1.8)	307	(1.8)	1.00
Liver cirrhosis	269	(1.6)	269	(1.6)	1.00
Peripheral vascular disease	123	(0.7)	123	(0.7)	1.00

CS, caesarian section; COPD, chronic obstructive pulmonary disease; PU, pressure ulcer.

*Surgical patients with and without decubitus ulcer were matched with propensity score by age, sex, low income, urbanization, operation in teaching hospital, type of surgery, type of anesthesia and coexisting medical conditions.

Compared with patients without pressure ulcer (Table 2), patients with pressure ulcer showed higher risks of postoperative complications, including pneumonia (RR 2.94, 95% CI 2.76–3.13), septicemia (RR 2.85, 95% CI 2.68–3.04), stroke (RR 2.02, 95% CI 1.88–2.17), urinary tract infection (RR 2.05, 95% CI 1.90–2.20), acute renal failure (RR 2.17, 95% CI 1.91–2.47), acute myocardial infarction (RR 1.45, 95% CI 1.12–1.87), and overall complications (RR 2.13, 95% CI 2.06–2.21). Preoperative pressure ulcer was associated with a significant increase in 30-day postoperative mortality (RR 1.83, 95% CI 1.54–2.18). Admission to ICU, prolonged length of hospital stay, and increased medical expenditures were all significantly associated with preoperative pressure ulcer, with respective RRs of 1.64 (95% CI 1.57–1.70), 2.73 (95% CI 2.58–2.88), and 2.03 (95% CI 1.94–2.14).

**Table 2:** Adverse events after surgeries in patients with preoperative pressure ulcer.

	No PU, %	PU, %	RR	(95% CI)*
Postoperative complications				
Pneumonia	7.4	21.8	2.94	(2.76–3.13)
Septicemia	7.4	21.1	2.85	(2.68–3.04)
Stroke	6.6	13.3	2.02	(1.88–2.17)
Urinary tract infection	6.3	12.9	2.05	(1.90–2.20)
Acute renal failure	2.0	4.3	2.17	(1.91–2.47)
Deep wound infection	0.9	1.1	1.21	(0.98–1.50)
Acute myocardial infarction	0.6	0.8	1.45	(1.12–1.87)
Postoperative bleeding	0.5	0.5	0.89	(0.67–1.20)
Pulmonary embolism	0.2	0.2	1.04	(0.61–1.78)
Any of the above	23.4	50.0	2.13	(2.06–2.21)
30-day in-hospital mortality	1.1	2.1	1.83	(1.54–2.18)
ICU stay	22.4	36.7	1.64	(1.57–1.70)
Prolonged length of hospital stay	10.3	28.1	2.73	(2.58–2.88)
Increased medical expenditure	13.2	26.8	2.03	(1.94–2.14)

CI, confidence interval; PU, pressure ulcer; ICU, intensive care unit; RR, rate ratio.

*Adjusted for age, sex, low income, urbanization, operation in teaching hospital, types of anesthesia, types of surgery and coexisting diseases.

The stratification analysis (Table 3) shows that the association between preoperative pressure ulcer and postoperative mortality was more significant in women (RR 1.87, 95% CI 1.43–2.46) than in men (RR 1.81, 95% CI 1.44–2.26). Surgical patients aged 70–79 years with pressure ulcer had the highest postoperative mortality of all age groups (RR 2.32, 95% CI 1.68–3.20).

**Table 3:** Stratification analysis by age, sex and coexisting medical conditions regarding association between preoperative pressure ulcer and postoperative mortality.

		30-day postoperative mortality			
	n	Deaths	Mortality, %	RR	(95% CI)*
Sex					
Female	15974	227	1.4	1.87	(1.43–2.46)
Male	18808	334	1.8	1.81	(1.44–2.26)
Age, years					
20–59	6672	68	1.0	1.62	(0.99–2.63)
60–69	4908	56	1.1	2.29	(1.30–4.06)
70–79	11690	176	1.5	2.32	(1.68–3.20)
≥80	11512	261	2.3	1.56	(1.22–2.00)

CI, confidence interval; RR, rate ratio.

*The rate ratio of postoperative mortality associated with pressure with multivariate adjustment.

Compared with surgical patients without pressure ulcer (Table 4), patients with pressure ulcer were more likely to have significantly higher postoperative mortality with debridement (RR 2.01, 95% CI 1.55–2.61), cellulitis (RR 1.89, 95% CI 1.48–2.41), wound (RR 2.07, 95% CI 1.65–2.60), and treatment by change dressing (RR 1.90, 95% CI 1.59–2.28), hospitalized care (RR 1.90, 95% CI 1.43–2.52), and antibiotics (with first-line antibiotics, RR 2.06, 95% CI 1.55–2.74; with second-line antibiotics, RR 3.74, 95% CI 2.33–5.99). The significant 30-day postoperative mortality compared with surgical patients without pressure ulcer was found in patients who had pressure ulcer with high total quantity of antibiotics (RR 3.46, 95% CI 2.33–5.15) and high medical expenditure on antibiotics (RR 2.60, 95% CI 1.88–3.60). Preoperative moderate-to-severe pressure ulcer was also associated with increased 30-day mortality after surgery (RR 1.92, 95% CI 1.61–2.29).

**Table 4:** Risk of 30-day postoperative in-hospital mortality associated with preoperative characteristics of pressure ulcer.

Characteristics of pressure ulcer within preoperative 24 months	30-day postoperative mortality				
	n	Deaths	Mortality, %	RR	(95% CI)*
Controls without pressure ulcer	17391	198	1.1	1.00	(reference)
Patients with pressure ulcer					
Antibiotics total quantity, mg†					
Low	15280	302	2.0	1.74	(1.46–2.08)
Moderate	706	14	2.0	1.74	(1.01–2.99)
High	701	19	2.7	2.26	(1.41–3.62)
Very high	704	28	4.0	3.46	(2.33–5.15)
Medical expenditure of antibiotics†					
Low	15264	302	2.0	1.75	(1.47–2.10)
Moderate	704	16	2.3	1.91	(1.15–3.19)
High	1408	45	3.2	2.60	(1.88–3.60)
Types of antibiotics					
Without antibiotics	14805	300	2.0	1.79	(1.50–2.14)
With first-line antibiotics (stage ≥2)‡	2586	63	2.4	2.06	(1.55–2.74)
With second-line antibiotics (stage ≥2)‡	412	19	4.6	3.74	(2.33–5.99)
Pressure ulcer with local infection					
No	14624	302	2.1	1.81	(1.51–2.16)
Yes (stage ≥2)‡	2767	61	2.2	1.97	(1.48–2.63)
Pressure ulcer with cellulitis					
No	12532	265	2.1	1.81	(1.51–2.18)
Yes (stage ≥2)‡	4859	98	2.0	1.89	(1.48–2.41)
Preoperative hospitalized care for PU					
No	13797	296	2.2	1.82	(1.52–2.18)
Yes (stage ≥2)‡	3594	67	1.9	1.90	(1.43–2.52)
Pressure ulcer with wound					
No	12236	241	2.0	1.73	(1.44–2.09)
Yes (stage ≥2)‡	5155	122	2.4	2.07	(1.65–2.60)
Pressure ulcer with debridement					
No	13728	284	2.1	1.79	(1.49–2.15)
Yes (stage ≥3)‡	3663	79	2.2	2.01	(1.55–2.61)
Pressure ulcer with change dressing					
No	4794	82	1.7	1.63	(1.26–2.12)
Yes (stage ≥2)‡	12597	281	2.2	1.90	(1.59–2.28)
Moderate-to-severe pressure ulcer§					
No (stage = 1)‡	2852	42	1.5	1.37	(0.98–1.91)
Yes (stage ≥2)‡	14539	321	2.2	1.92	(1.61–2.29)

CI, confidence interval; PU, pressure ulcer; RR, rate ratio.

*Adjusted for age, sex, low income, urbanization, coexisting medical conditions, operation in teaching hospital, types of anesthesia and types of surgery.

^†^Antibiotics total quantity was categorized into low (non-antibiotics users and lowest quartile), moderate (second quartile), high (third quartile), and very high (highest quartile). Medical expenditure of antibiotics was categorized into low (non-antibiotics users and lowest quartile), moderate (second quartile), and high (third quartile and highest quartile)

^‡^Based on the criteria of The National Pressure Ulcer Advisory Panel, the stage of pressure ulcer was categorised by clinical physicians in Taiwan and previous investigations.

^§^Patients with pressure have at least one of characteristics, such as debridement, local infection, cellulitis, wound, change dressing, use of antibiotics, and hospitalized care.

## Discussion

This nationwide population-based, propensity score-matched cohort study showed that patients with preoperative pressure ulcer had increased risk of postoperative 30-day in-hospital mortality and complications such as septicemia, pneumonia, stroke, urinary tract infection and acute renal failure. Patients with pressure ulcer receiving debridement, or having local infection, cellulitis or treatment with antibiotics had significantly higher 30-day postoperative mortality. Postoperative admission to intensive care unit, prolonged length of hospital stay and increased medical expenditures were also associated with preoperative pressure ulcer. To our knowledge, this investigation is the first study investigating adverse events after surgery in patients with preoperative pressure ulcer.

Although the association between preoperative pressure ulcer and postoperative adverse outcomes was still unclear, previous studies had identified factors associated with postoperative pressure ulcer such as old age, types of surgeries and coexisting medical conditions [17,18]. These factors were also associated with major adverse events after surgery [14–16,19–25]. To reduce the confounding errors, we adjusted these covariates using propensity score-matched procedure and then controlled these factors in the multivariate Poisson regression models. In the present study, preoperative pressure ulcer was associated with postoperative mortality in both sexes and all age groups, confirming the association between pressure ulcer and postoperative mortality.

The National Pressure Ulcer Advisory Panel had proposed a classification system to grade pressure ulcer from stage I to stage IV according to the integrity of epidermis and the involved depth of wound [26]. As skin defects provide entrances for bacteria to colonize, invade and infect the underlying tissues, local or systemic infection is the most prevalent complication of pressure ulcers presenting open wounds (stage II-IV), and debridement is indicated for such infected necrotic tissues [27]. As a result, coexisting local infection of skin implied more advanced pressure ulcer, as did performance of debridement, abscess and cellulitis, particularly in those using second-line antibiotics and with higher total quantities of antibiotics and higher expenditures on antibiotics. Our study found that such advanced pressure ulcers were associated with significantly higher postoperative mortality.

Some possible explanations may clarify the association between preoperative pressure ulcer and postoperative adverse outcomes. First, the major predisposing factor of pressure ulcer is immobilization, which may result from stroke, spinal cord injury, progressive neurologic disorders or hip fracture. Immobilization also impairs the ability to clear bronchial secretions, and can eventually result in atelectasis and hypostatic pneumonia [28]. Second, pressure ulcer is one of causes several complications, including localized abscess, cellulitis, bacteremia, sepsis, osteomyelitis, fistula and carcinoma [27]. Among these complications, infection resulting from polymicrobial organism is the most prevalent; such infection can be life-threatening when complicated by bacteremia, sepsis, endocarditis and meningitis [27,29–31]. Infected pressure ulcers have been independently associated with bloodstream infection as well as with higher 30-day mortality in hospitalized patients [32]. Third, chronic pressure ulcer features persistent chronic inflammation with leukocyte infiltration and release of proinflammatory cytokines [33,34]. Repetitive pressure and relief also lead to cutaneous ischemia-reperfusion injury, causing infiltration of inflammatory cells, deleterious cellular reactions and tissue damage by reactive free oxygen radicals [35]. Extensive evidence shows that chronic inflammation and infection outside the brain are associated with an increased risk of ischemic stroke via endothelial cell dysfunction, impaired fibrinolysis and activation of platelet aggregability altered by immune-mediated inflammatory cells and cytokines [36–39]. These biochemical cascades in pressure ulcer lead to repetitive chronic inflammation, endothelial cell dysfunction and microvasculature system thrombosis that may contribute to stroke and ischemic renal injury. Finally, presence of pressure ulcers may indicate inadequate services, poor nutrition or insufficient family support for the patients; these factors might indirectly contribute to poor surgical outcomes and higher mortality. Regarding nutritional deficiency in particular, fewer scavengers to remove free oxygen radicals may result in reactive vasoconstriction and increased cell damage.

Prevention of pressure ulcer is always an important issue for hospitalized patients requiring coordination among multidisciplinary medical teams. We suggest that assessment of risk of developing pressure ulcer and grading of existing pressure ulcer are essential for surgical patients. Patients with serious or infected pressure ulcer must receive adequate wound dressing, debridement of necrotic tissues and early empiric antimicrobial therapy preoperatively to reduce potential postoperative mortality. Pressure ulcer patients receiving surgery require appropriate monitoring and adequate control of coexisting medical conditions such as cardiovascular disease, diabetes mellitus and neurogenic degenerative disease. Due to the documented correlation between nutritional status and pressure ulcer [40], adequate protein, vitamins and mineral supplements should be provided for patients with pressure ulcer.

There are some limitations in this study. First of all, detailed information on patient sociodemographics, lifestyle, nutritional status, biomedical measurements, and the clinical stage and causes of pressure ulcer was not available from the National Health Insurance Research Database. However, we added some information regarding the possible stage of pressure in the tables according to previous investigations [41–45]. Second, because this study lacks detailed grading of preoperative pressure ulcer [26], we could not investigate the impact of severity of pressure ulcer on postoperative mortality. In addition, as our study used ICD-9-CM codes to define coexisting medical conditions and postoperative complications, miscoding due to human error could not be avoided; however, the accuracy of the National Health Insurance Research Database has been accepted in numerous scientific publications [13–16,24]. Finally, possibly inadequate adjustment of confounding factors in use of propensity score-matched pair technique might cause neglected bias in the study. Although we controlled for several confounders, residual confounding is always possible.

This nationwide, propensity score-matched, retrospective cohort study is the first report investigating increased adverse events after surgery in patients with preoperative pressure ulcer. We suggest that preoperative pressure ulcers may be a marker for more severely morbid patients who tolerate surgery more poorly.
